# Optimization of Mechanical Properties of Multiphase Materials with Auxetic Phase

**DOI:** 10.3390/ma19010103

**Published:** 2025-12-27

**Authors:** Maciej Zawistowski, Arkadiusz Poteralski

**Affiliations:** Department of Computational Mechanics and Engineering, Faculty of Mechanical Engineering, Silesian University of Technology, Konarskiego 18A, 44-100 Gliwice, Poland; arkadiusz.poteralski@polsl.pl

**Keywords:** auxetic materials, finite element method, multiphase material, multiscale modeling, numerical simulation, parametric optimization

## Abstract

Auxetic materials and structures exhibit negative values of Poisson’s ratio, which is the source of their unusual deformation pattern. Auxetic materials can be utilized in the development of multiphase materials with increased Young’s modulus by properly distributing the different phases in the volume of composite material and utilizing the auxetic effect. This work presents the results of an optimization of multiphase materials with an auxetic phase, with the aim of obtaining increased stiffness and near-zero lateral strain. Geometries of auxetic unit cells and conventional unit cells were subjected to optimization to obtain the desired values of effective material properties via multiscale modelling. Values of material properties of all considered phases were obtained via multiscale modelling of representative volume elements of their respective auxetic and conventional unit cells. Four types of unit cells and three types of inclusion patterns in the hybrid material sample were considered. The simulation results demonstrate that the application of an auxetic phase region in the multiphase material allows it to obtain effective Young’s modulus greater than that of component phases, as well as near-zero lateral strain during uniaxial tension of the sample. Increase of effective Young’s modulus and significant reduction of effective Poisson’s ratio of the sample were obtained in all considered optimization cases.

## 1. Introduction

Since 1991, materials with negative Poisson’s ratio *ν* have been known as auxetics. The term is derived from Greek αÙξητικo’ς (auxetikos), meaning “that which tends to increase” and was proposed by Evans [1]. Three main deformation patterns of bodies subjected to uniaxial tension can be distinguished:

- For *ν* > 0, the body elongates in the direction of tension and shrinks laterally. This is the most common behavior observed in conventional materials.
- For *ν* < 0, the body both elongates in the direction of tension and expands laterally. This counter-intuitive behavior is referred to as “auxetic”.
- For *ν* = 0, a special case, the body only elongates in the direction of tension and its lateral dimensions do not change. An example of natural material with near-zero Poisson’s ratio is cork [2].

**Figure 1:**
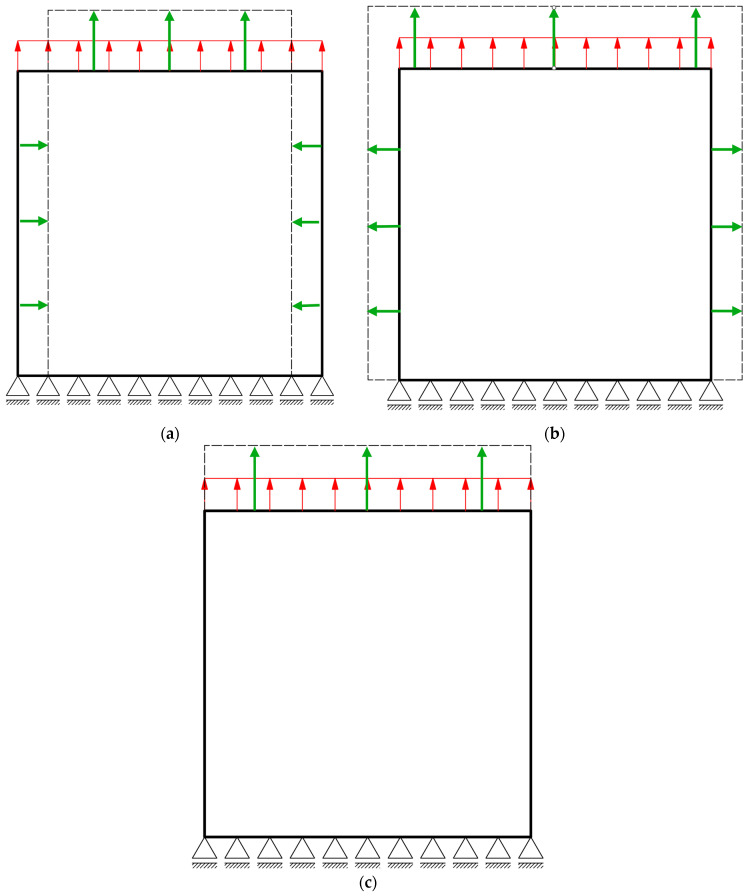
Types of materials deformation pattern during uniaxial tension. Solid line—contour of the undeformed sample; dashed line—contour of the deformed sample; green arrows—strain, red arrows—uniform load; (**a**) conventional material, (**b**) auxetic material, (**c**) zero-value Poisson’s ratio material.

Schematic illustration of deformation patterns of different types of materials subjected to uniaxial tension is given in Figure 1.

The first reported observations of auxetic behavior come from Love in 1927 [3] and Voigt in 1928 [4], who both observed peculiar behavior of pyrite crystals during experiments. Love described an example of single crystal pyrite with negative Poisson’s ratio of −0.14. Auxetics as a research topic resurged in the 1980s, when synthetic foam with negative Poisson’s ratio was manufactured. Bhullar describes this period in his review of auxetics research [5].

Auxetic behavior is a direct effect of the internal structures’ geometry. In macroscale auxetic structures, the deformation pattern similar to an action of linkage mechanism can be directly observed. In case of auxetic materials, these deformations happen in microscale with deformation of unit cells. Conventional materials can be used to obtain auxetic structures and materials via shaping them into auxetic geometries [6,7,8]. Multiscale modeling is often utilized in order to obtain the auxetic material properties based on the geometry of a specific unit cell [9,10]. Researchers focus on singular unit cells in simulations in order to obtain auxetic materials with desired properties [11,12,13].

Unusual deformation patterns and effective material properties of auxetics can be utilized in many interesting applications. An example of composite material combining auxetic and conventional components in order to obtain effective zero-value Poisson’s ratio was proposed by Evans and Alderson for a bullet with reduced friction during movement in the barrel [7]. The numerous applications of auxetics considered in the literature include personal protection, bulletproof vests, cementitious composites, and crash boxes, among others [14,15,16,17,18,19,20,21,22].

As auxetics are rarely characterized with high stiffness in comparison to conventional materials, research often concentrates on increasing the auxetics’ stiffness [23]. Auxetic behavior can also be obtained by combining anisotropic structures which do not behave auxetically on their own [24]. Approach of combining phases with different material properties into a multimaterial or a hybrid material, is an underlying principle in development of modern composites with custom-tailored properties [25,26].

Long et al. utilized the auxetic effect in a two-phase composite material to maximize the effective Young’s modulus. In their study, they used phases with material properties of Poisson’s ratio equal to 0.4 and −0.9, while the Young’s modulus, depending on the case, was either equal to 1.0 for both cases or was greater for the auxetic phase and ranged from 3.0 to 9.0 [27]. Their previous research indicates that auxetics in most cases have significantly lower stiffness than conventional phases, especially auxetics with a strong auxetic effect [28,29]. In this study, material properties were determined based on multiscale modeling of auxetic geometries. One of the geometries was optimized to maximize the effective Young’s modulus of the unit cell.

Metamaterials with zero Poisson’s ratio, like auxetics, offer unique advantages due to their unconventional deformation pattern. They can be used in applications requiring dimensional stability. Their potential applications include vibration control, biomimic scaffolds for cartilage or ligament tissue, energy absorption systems, and morphing wings. Development of zero Poisson’s ratio metamaterials is an active research topic. For example, an isotropic zero Poisson’s ratio metamaterial based on the aperiodic monotile has been proposed [30], unique in the aspect of using a structure composed of a single type of unit cell, forming a mosaic-like pattern of continuously rotated ‘hats’. Spring- or helical-based 3D lattice metamaterials are another example [31]. A more traditional approach is to combine structures with both positive and negative Poisson’s ratios, so that the resulting metamaterial has a zero-value Poisson’s ratio. An example of such approach is the AUXHEX Kirigami-inspired cellular structure [32].

In this paper, we propose an approach to developing near-zero Poisson’s ratio metamaterials with increased stiffness, based on parametric optimization of regular-shaped inclusion regions in the multimaterial sample. While four example unit cells are considered, the same methodology can be applied to different types of unit cells. The results of optimization of multiphase materials with conventional and auxetic phases, with the goal to obtain simultaneously near-zero effective Poisson’s ratio and higher effective Young’s modulus than component phases, are presented. Multiscale modeling and FEM simulations coupled with parametric optimization have been carried out with the use of Ansys Workbench Mechanical 2024 R1 software.

## 2. Materials and Methods

The term “effective material properties” in the context of this paper means that we do not consider the material properties of the bulk material of the structure, but rather the effective properties of the structure itself, treating the unit cell and the sample as if they were a new, separate material with different properties than the bulk material of which they are actually composed. This approach is often used in the context of auxetic materials and auxetic structures research [6,8]. Stress, strain, and Young’s modulus are fundamental concepts in strength of materials; their respective definitions, formulas, and derivations can be found in many handbooks, e.g., in [33]. Effective Young’s modulus and effective Poisson’s ratio are determined based on the deformation of the external edges of the structure which connect it to the neighboring unit cells.

The effective strain of the sample in the considered direction can be expressed as:

(1) $$\varepsilon_{eff}=\frac{{∆L}_{avg}}{L},$$

where $\varepsilon_{eff}$ denotes effective strain, L is the initial total length of the sample, and ${∆L}_{avg}$ is the averaged increment of length, measured on the sample’s external edges.

The effective Poisson’s ratio is calculated as:

(2) $$\upsilon_{eff}=-\frac{\varepsilon_{Teff}}{\varepsilon_{Aeff}},$$

where $\varepsilon_{Teff}$ denotes effective transversal strain and $\varepsilon_{Aeff}$ the effective axial strain.

The effective stress is equal to the loading force, *P*, divided by the sample’s cross-section area, *A*, as follows:

(3) $$\sigma_{eff}=\frac{P}{A}.$$

The effective Young’s modulus is determined based on the effective stress and effective strain in the direction of the loading force as follows:

(4) $$E_{eff}=\frac{\sigma_{eff}}{\varepsilon_{Aeff}}.$$

### 2.1. Multiscale Modeling

Multiscale modeling was applied in order to obtain the material properties of the phases composed of considered unit cells. Ansys Material Designer software [34] was used in order to obtain the material properties based on representative volume elements of microscale unit cells geometries. Orthotropic anisotropy was considered and periodic boundary conditions were applied. The process was similar to that applied in a previous study, where it was described in depth [29].

Four different types of unit cells were considered; auxetic hex reentrant, rotating rectangles unit, conventional uniform honeycomb, and orthogonal grid. First, the material properties of conventional unit cells were determined. Then the auxetic unit cells were subjected to initial optimization in order to obtain comparable values of stiffness and density to the conventional unit cells. Then, the obtained materials were paired based on their effective Young’s modulus to be used as component phases. The process of parametric optimization of auxetic unit cells in order to obtain desired effective material properties by changing the geometry was described in detail in a previous study [28].

All unit cells had bulk size equal to 20 μm. A maximum mesh size of 0.2 μm was assumed. ABS polymer was selected as the bulk material; the unit cells had bulk material properties of Young’s modulus equal to 1.628 GPa and Poisson’s ratio equal to 0.4089, with density equal to 1030 kg/m^3^.

**Figure 2:**
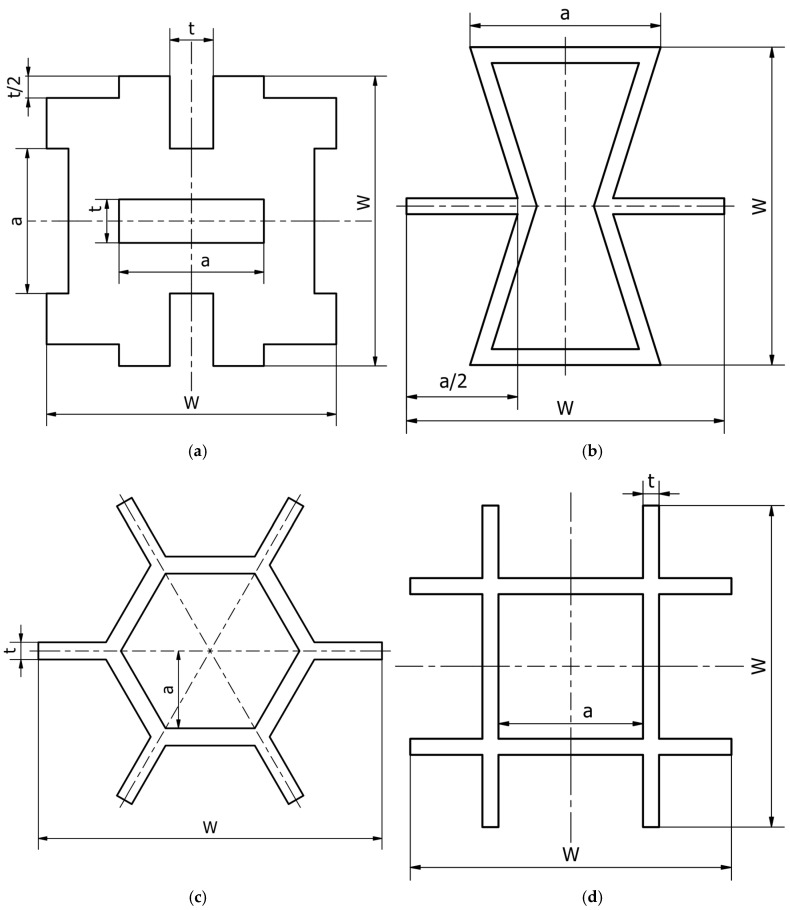
Considered unit cells: (**a**) rotating rectangles unit, (**b**) hex reentrant, (**c**) uniform honeycomb, (**d**) orthogonal grid; W, a, t—geometrical parameters as per Table 1. All unit cells have the same bulk dimensions, W × W equal to 20 μm.

The parametrized unit cells considered in this work are shown in Figure 2. The final dimensions of the unit cells post initial optimization are given in Table 1. The effective material properties of the unit cells are given in Table 2.

### 2.2. Multiphase Material

**Figure 3:**
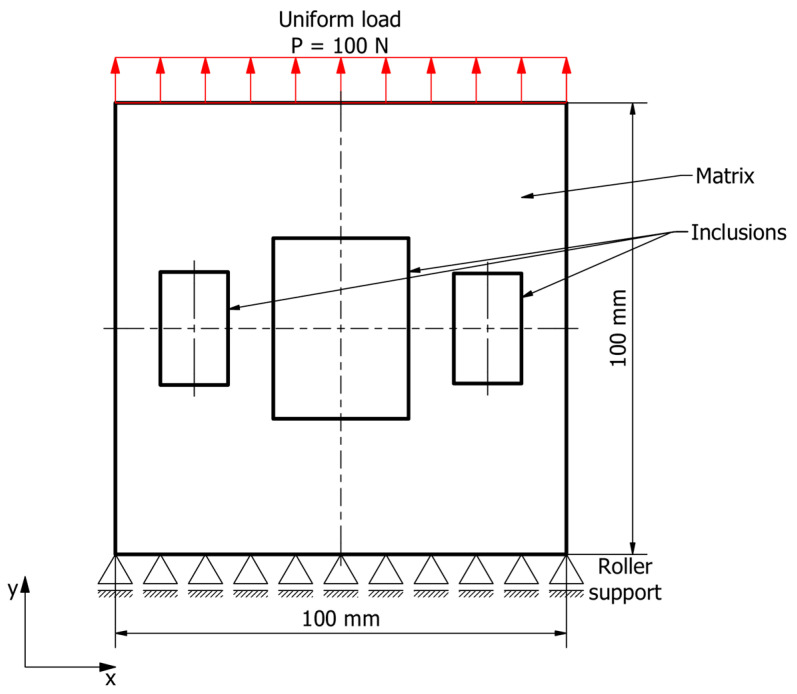
Sample schematic with boundary conditions.

A 100 × 100 mm square sample of hybrid material with rectangular inclusion regions was considered. Two-dimensional finite element analysis with sample thickness equal to 1 mm was conducted. The bottom edge of the sample was supported by rollers and the top edge of the sample was subject to a uniform tensile load of 100 N. A 0.5 mm-sized uniform quadrilateral finite element mesh was applied. The boundary conditions and sample dimensions are given in Figure 3.

Since the bulk dimensions of the sample and the magnitude of the load were constant, the effective stress in the sample was also constant and equal to 1 MPa:

(5) $$\sigma_{eff}=\frac{P}{A}=\frac{100\;N}{100\;mm^{2}}=1\;MPa.$$

The inclusion regions were parametrized and distributed symmetrically. Parameter constraints were applied, so that there would always be at least 2.5 mm of the matrix material between the inclusion regions and the external edges of the sample.

Cases where the inclusions were the auxetic phase and the matrix was the conventional phase, as well as where the inclusions were conventional and matrix was auxetic, were both considered for all inclusions’ distribution patterns.

**Figure 4:**
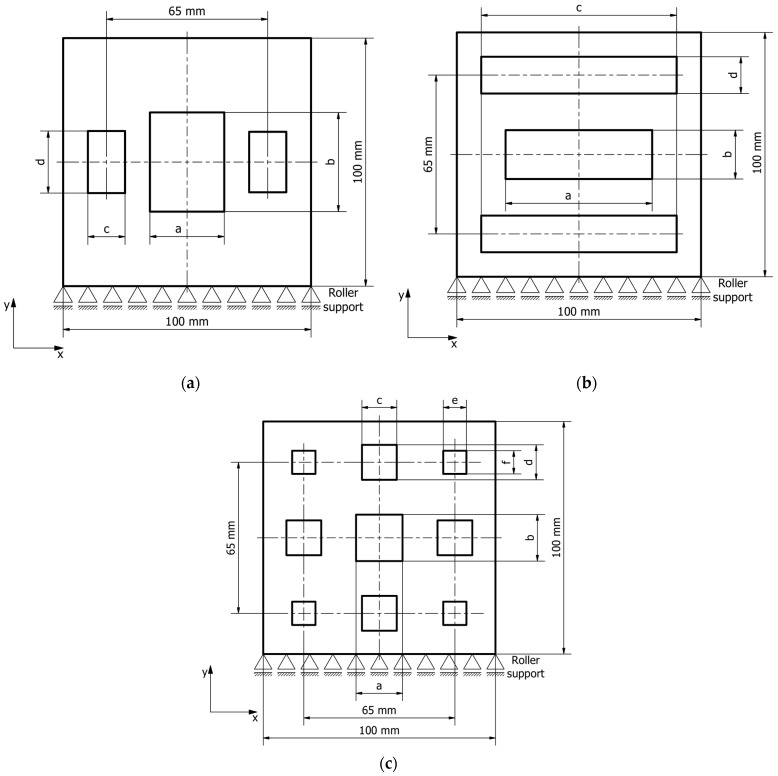
Considered inclusions patterns: (**a**) 3 vertical rectangles, (**b**) 3 horizontal rectangles, (**c**) 9 rectangles.

The first inclusion pattern consists of three vertical rectangles. The center rectangle is independent, while the outer rectangles are symmetrical to each other. There are four independent geometrical parameters. The second inclusion pattern is analogous to the first, but the rectangles are oriented horizontally. The third inclusion pattern is more complex, consisting of nine symmetrically distributed rectangles; the center rectangle is independent, the four rectangles in the corners are equal to each other, and the four rectangles near the sample edges’ midpoints are also equal to each other, resulting in a total of six independent parameters. The considered patterns of inclusions distribution are given in Figure 4.

A complete list of all considered cases of combinations of the inclusion patterns and inclusion and matrix materials is given in Table 3.

### 2.3. Optimization

Ansys Response Surface Optimization was used in the process of identifying the values and geometrical parameters of the inclusion regions which would allow us to obtain the desired increase in stiffness and near-zero Poisson’s ratio. A Multi Objective Genetic Algorithm was applied in the optimization. The optimization function had two simultaneous objectives: first, to maximize the effective Young’s modulus in the axial load direction, and second, to bring the value of effective Poisson’s ratio to zero:

*E_yeff_* (*a*, *b*, *c*, *d*, *e*, *f*) ⇒ *max* (6)

*ν_eff_* (*a*, *b*, *c*, *d*, *e*, *f*) ⇒ 0, (7)

The optimization range of geometric parameters for considered inclusion distribution patterns is given in Table 4.

The optimization settings parameters were as follows:

- Number of initial samples—6000
- Number of samples per iteration—1200
- Maximum allowable Pareto percentage—70
- Convergence stability percentage—2
- Maximum number of iterations—20
- Maximum number of candidates—20

In all considered cases, all of the generated candidates were verified via additional numerical analysis to confirm the results.

## 3. Results

### 3.1. 3 Vertical Rectangles

**Figure 5:**
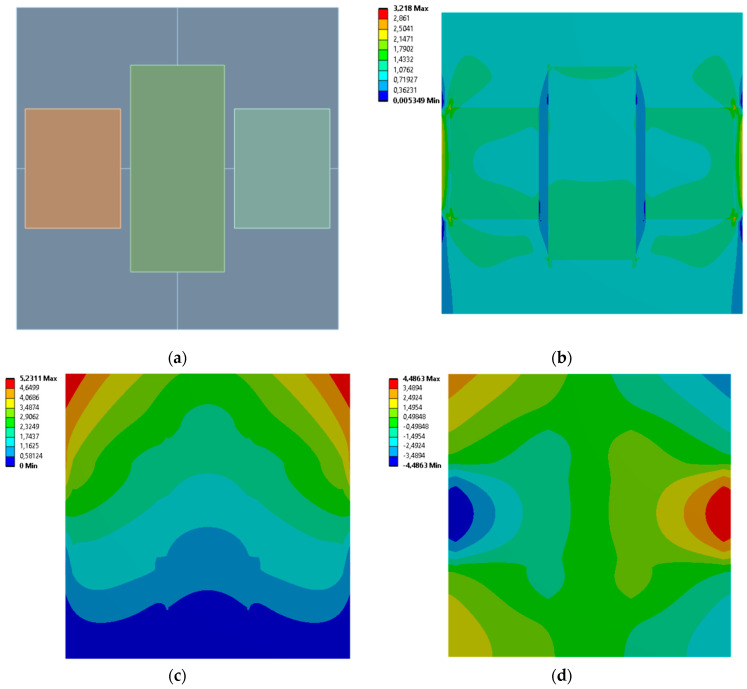
Case 1 results; (**a**) geometry of the sample, (**b**) equivalent stress (Huber-von Mises-Hencky) [MPa], (**c**) vertical deformation [mm], (**d**) horizontal deformation [mm].

**Figure 6:**
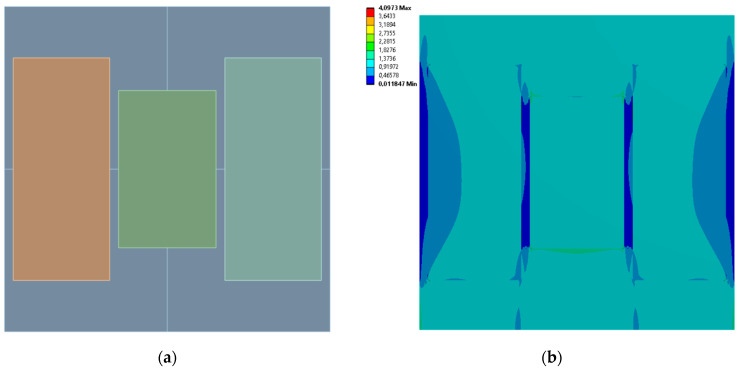

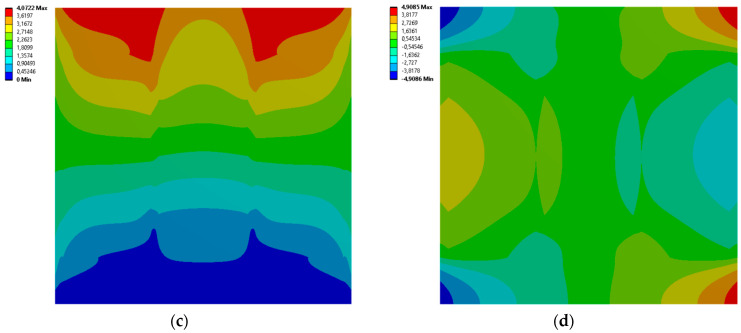
Case 2 results; (**a**) geometry of the sample, (**b**) equivalent stress (Huber-von Mises-Hencky) [MPa], (**c**) vertical deformation [mm], (**d**) horizontal deformation [mm].

**Figure 7:**
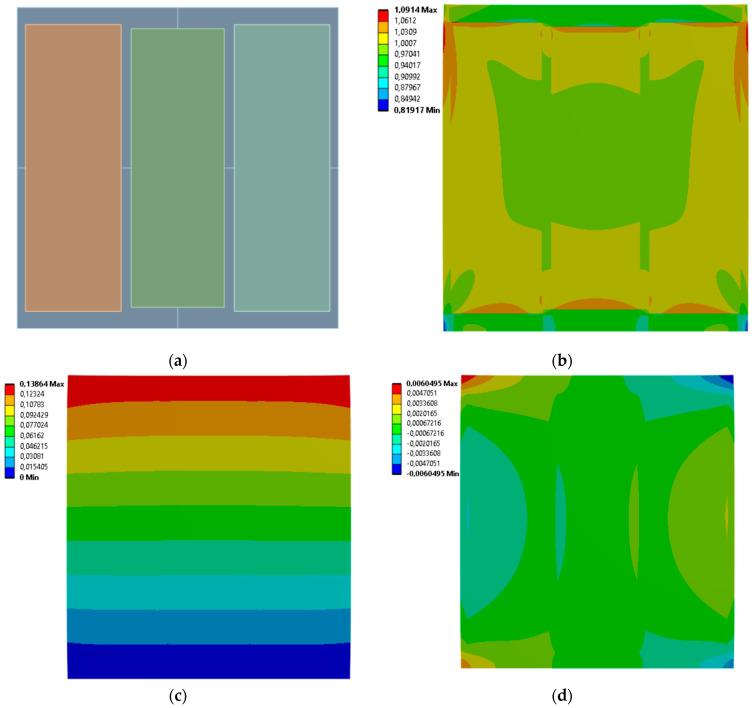
Case 3 results; (**a**) geometry of the sample, (**b**) equivalent stress (Huber-von Mises-Hencky) [MPa], (**c**) vertical deformation [mm], (**d**) horizontal deformation [mm].

**Figure 8:**
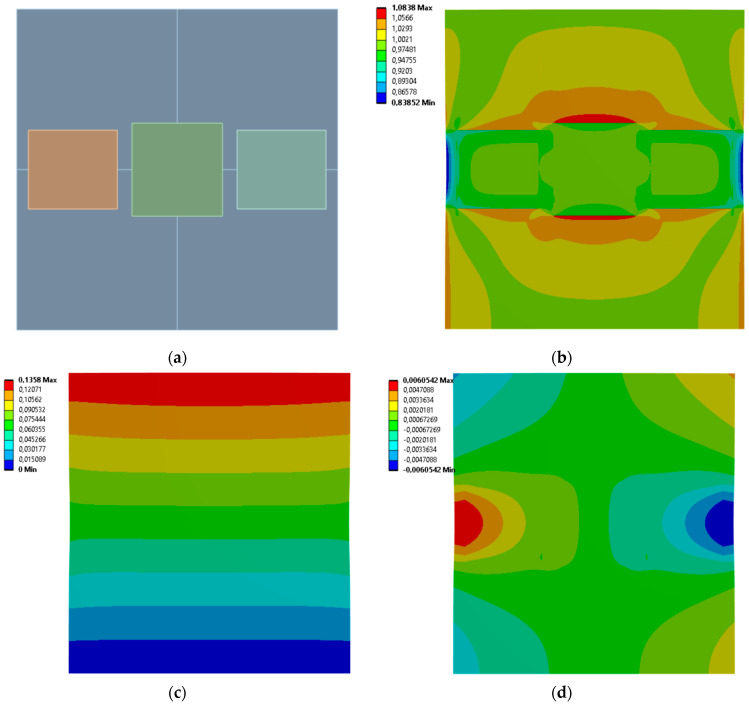
Case 4 results; (**a**) geometry of the sample, (**b**) equivalent stress (Huber-von Mises-Hencky) [MPa], (**c**) vertical deformation [mm], (**d**) horizontal deformation [mm].

Figure 5, Figure 6, Figure 7 and Figure 8 show equivalent stress and directional deformations of Cases 1–4, samples with 3 vertical rectangles inclusion distribution pattern. The inclusion dimensions and the results of effective Young’s modulus and effective Poisson’s ratio of the samples are given in Table 5.

### 3.2. 3 Horizontal Rectangles

**Figure 9:**
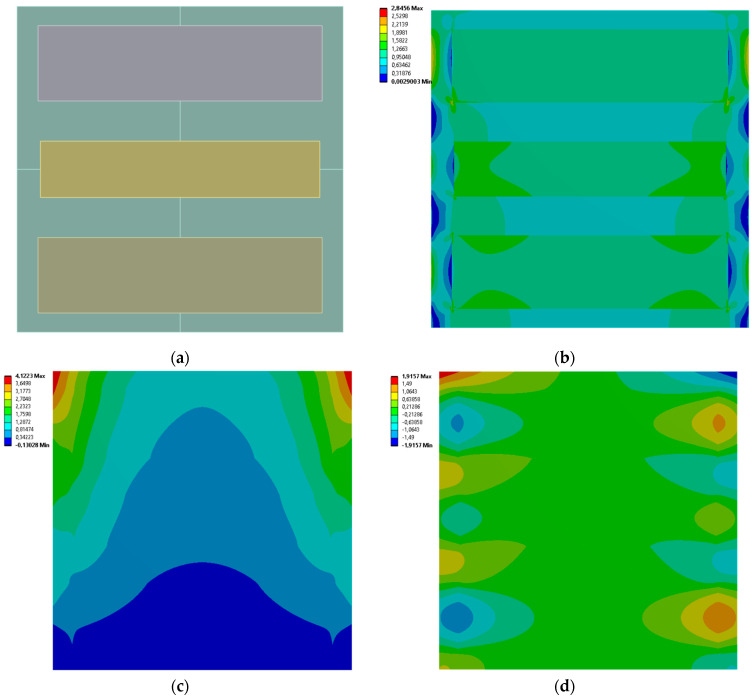
Case 5 results; (**a**) geometry of the sample, (**b**) equivalent stress (Huber-von Mises-Hencky) [MPa], (**c**) vertical deformation [mm], (**d**) horizontal deformation [mm].

**Figure 10:**
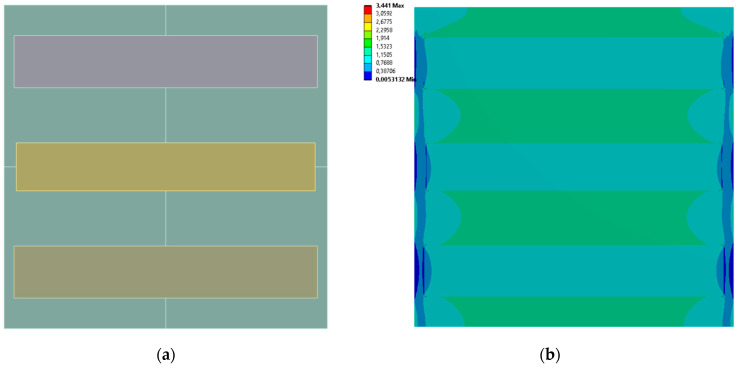

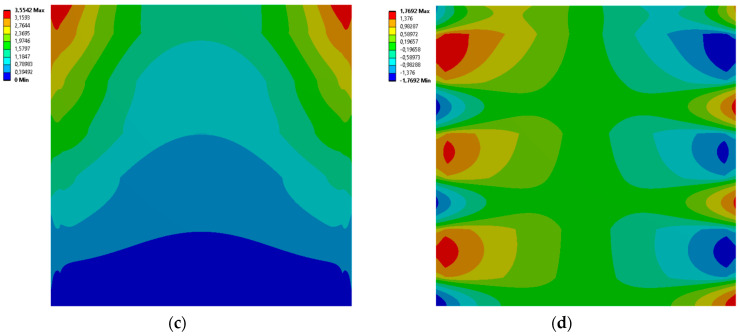
Case 6 results; (**a**) geometry of the sample, (**b**) equivalent stress (Huber-von Mises-Hencky) [MPa], (**c**) vertical deformation [mm], (**d**) horizontal deformation [mm].

**Figure 11:**
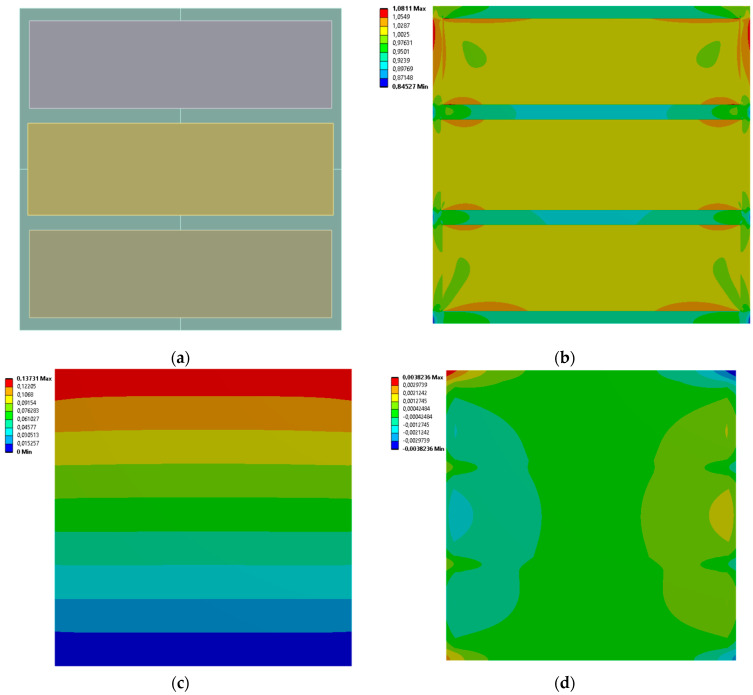
Case 7 results; (**a**) geometry of the sample, (**b**) equivalent stress (Huber-von Mises-Hencky) [MPa], (**c**) vertical deformation [mm], (**d**) horizontal deformation [mm].

**Figure 12:**
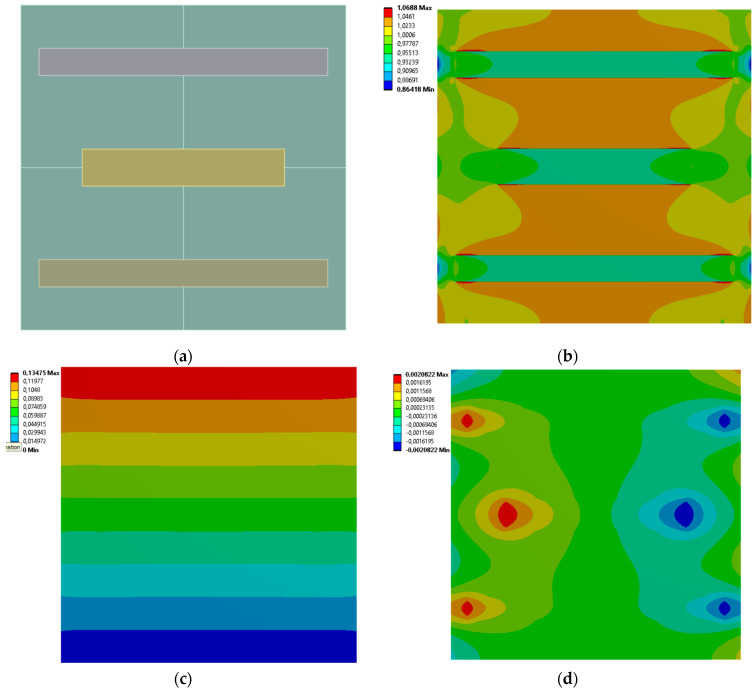
Case 8 results; (**a**) geometry of the sample, (**b**) equivalent stress (Huber-von Mises-Hencky) [MPa], (**c**) vertical deformation [mm], (**d**) horizontal deformation [mm].

Figure 9, Figure 10, Figure 11 and Figure 12 show equivalent stress and directional deformations of Cases 5–8, samples with 3 horizontal rectangles inclusion distribution pattern. The inclusion dimensions and the results of effective Young’s modulus and effective Poisson’s ratio of the samples are given in Table 6.

### 3.3. 9 Rectangles

**Figure 13:**
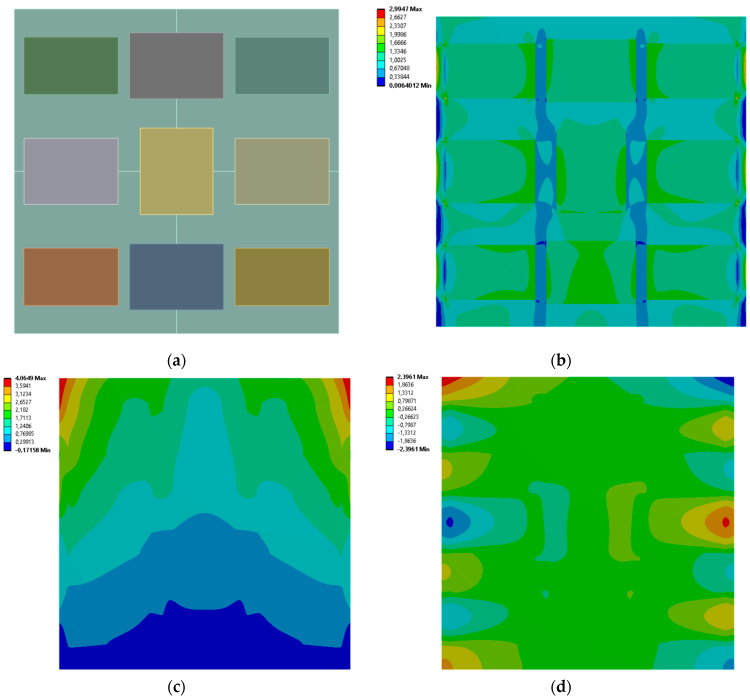
Case 9 results; (**a**) geometry of the sample, (**b**) equivalent stress (Huber-von Mises-Hencky) [MPa], (**c**) vertical deformation [mm], (**d**) horizontal deformation [mm].

**Figure 14:**
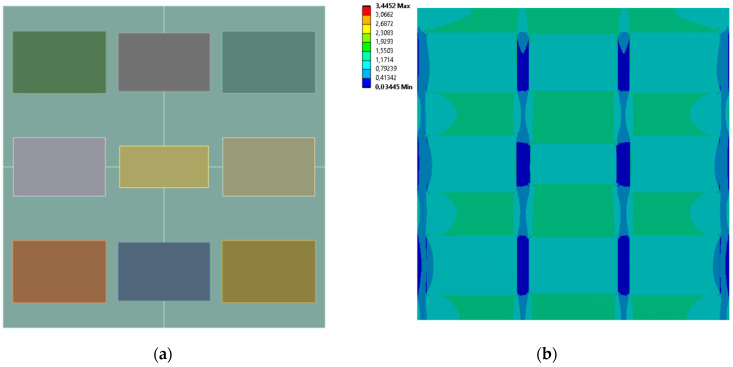

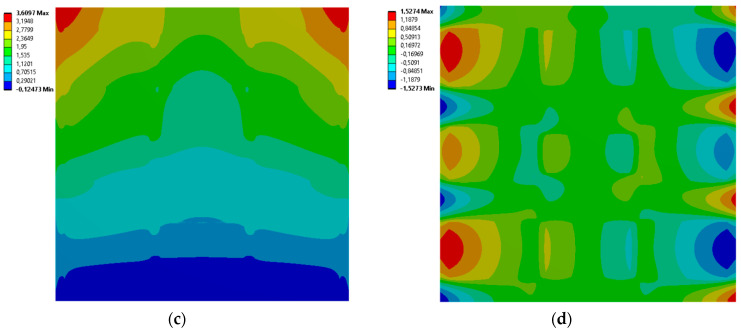
Case 10 results; (**a**) geometry of the sample, (**b**) equivalent stress (Huber-von Mises-Hencky) [MPa], (**c**) vertical deformation [mm], (**d**) horizontal deformation [mm].

**Figure 15:**
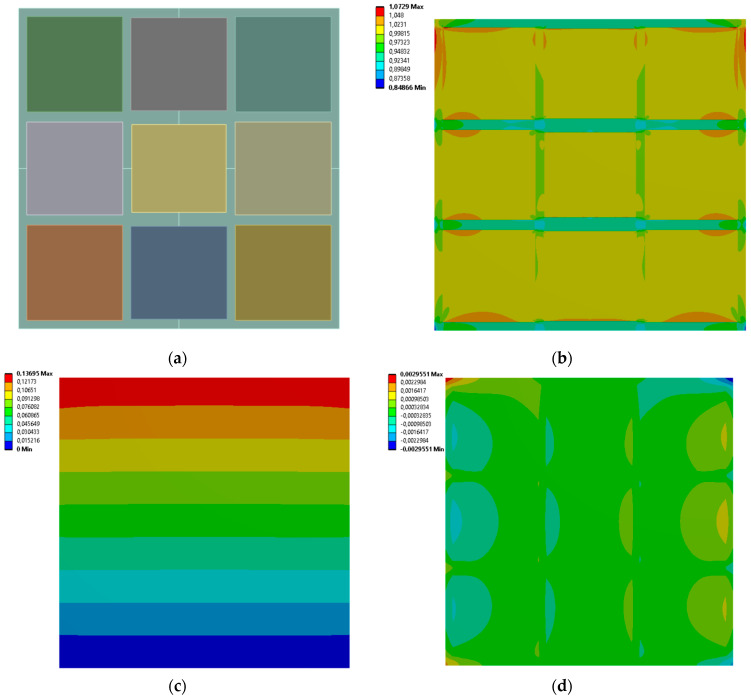
Case 11 results; (**a**) geometry of the sample, (**b**) equivalent stress (Huber-von Mises-Hencky) [MPa], (**c**) vertical deformation [mm], (**d**) horizontal deformation [mm].

**Figure 16:**
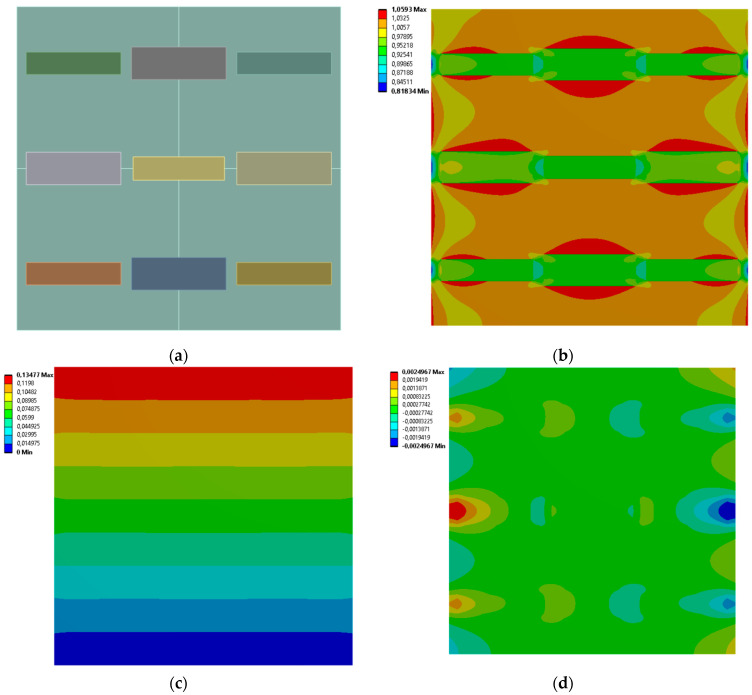
Case 12 results; (**a**) geometry of the sample, (**b**) equivalent stress (Huber-von Mises-Hencky) [MPa], (**c**) vertical deformation [mm], (**d**) horizontal deformation [mm].

Figure 13, Figure 14, Figure 15 and Figure 16 show the equivalent stress and directional deformations of Cases 9–12, samples with 9 rectangles inclusion distribution pattern. The inclusion dimensions and the results of effective Young’s modulus and effective Poisson’s ratio of the samples are given in Table 7.

## 4. Discussion

The obtained results show that it is possible to obtain a hybrid material with both increased stiffness and near-zero effective Poisson’s ratio. In the case of both considered pairs, the most significant improvement of effective Young’s modulus was obtained for the “3 horizontal rectangles” inclusion distribution pattern. While for the rotating rectangles unit/orthogonal grid pair, the improvement was miniscule (maximum of 742.090 MPa compared to 740.260 Young’s modulus of the component phase, which is an improvement of 0.247%), for the hex reentrant/uniform honeycomb pair, the improvement was significant (maximum of 56.365 MPa compared to 20.980 MPa, which is an improvement of 168.661%). The condition of obtaining near-zero effective Poisson’s ratio was fulfilled by both pairs; for the rotating rectangles unit/orthogonal grid pair, very small values in the range of 10^−4^ and 10^−6^ were obtained and the maximal reduction in comparison to −0.046 initial value is equal to over 4700 times. For the hex reentrant/uniform honeycomb pair, the smallest value was 0.016 and, compared to initial value of −0.329, the maximal reduction was 20.5 times.

By comparing the deformation distributions, we can see that the lateral edges of the samples are not uniformly deformed. The strong irregularities are most significant in the “3 vertical rectangles” inclusion distribution pattern. For the “3 horizontal rectangles” and “9 rectangles” inclusion distribution patterns, the irregularities are also visible, but overall, the deformation pattern is much more uniform.

While a significant increase in effective Young’s modulus was obtained, the resulting metamaterials are characterized with stress concentration. The phenomenon is most visible in the hex reentrant/uniform honeycomb pair. Compared to the uniform stress for homogenous sample equal to 1 MPa (result of 100 N load acting on 100 mm^2^ cross-section area), the stress concentration is up to four times higher. This is a direct result of the auxetic effect and interfacing of phase regions with significantly differing effective Poisson’s ratios. Moreover, the stress concentration is highly localized, which is a consequence of sharp edges on the corners of the inclusion regions. This effect could be reduced by applying fillets.

The next stage of research could focus on experimental validation of these results, with upscaled 3D-printed or machined samples. Further exploration of possible improvement of the proposed approach could also be made by applying it to different types of unit cells, inclusion distribution patterns or more complex sample geometries and boundary conditions.

The results show that combining two phases with significantly differing Poisson’s ratios, one strongly conventional and one strongly auxetic, it is possible to obtain very significant improvement of the effective Young’s modulus and a near-zero Poisson’s ratio. Combining a conventional phase with a near-zero Poisson’s ratio does not produce a significant improvement in effective Young’s modulus, but does make it possible to obtain a true near-zero effective Poisson’s ratio.

## 5. Conclusions

This paper presents the results of an optimization of multiphase materials with conventional and auxetic phases with the goal of obtaining simultaneously near-zero effective Poisson’s ratio and higher effective Young’s modulus than component phases. Three different types of inclusion patterns were considered, and analyses were conducted for cases of auxetic inclusions and conventional matrices as well as conventional inclusions and auxetic matrices. A total of twelve cases were presented in the results, proving the initial assumption that it is possible to obtain near-zero effective Poisson’s ratio and increased effective Young’s modulus by combining two phases with significantly different effective Poisson’s ratios and comparable stiffness.

The component phases were paired based on their stiffness and density. While both pairs exhibited simultaneous improvement of stiffness and near-zero Poisson’s ratio, the improvement of stiffness was much more significant for the pair of phases with significantly different values of Poisson’s ratio. A consistent increase of the sample’s effective Young’s modulus was obtained simultaneously with a significant reduction of effective Poisson’s ratio to near-zero values. A significant increase in stiffness was also obtained (up to 168% compared to component phases) simultaneously with considerable reduction of effective Poisson’s ratio (down to 0.016).

While four specific types of unit cells were considered, the same approach can be used with different geometries.

## Figures and Tables

**Table 1:** Dimensions of considered unit cells.

Unit Cell	W [μm]	a [μm]	t [μm]
Conventional honeycomb	20	4.5	1
Hex reentrant	20	16	1
Orthogonal grid	20	6	4
Rotating rectangles unit	20	10	3

**Table 2:** Effective material properties of considered unit cells.

**Phase type** **Unit cell**	**Conventional** **Uniform honeycomb**	**Auxetic** **Hex reentrant**
Geometry	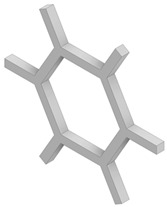	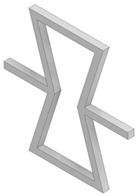
Density *ρ* [kg/m^3^]	165.890	195.820
Young’s modulus *E_x_* [MPa]	10.566	2.192
Young’s modulus *E_y_* [MPa]	20.980	17.135
Poisson’s ratio *ν*	0.650	−0.329
**Phase type** **Unit cell**	**Conventional** **Orthogonal grid**	**Auxetic** **Rotating rectangles unit**
Geometry	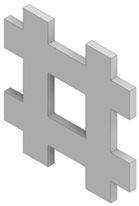	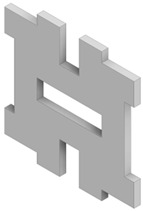
Density *ρ* [kg/m^3^]	659.200	929.400
Young’s modulus *E_x_* [MPa]	732.060	740.260
Young’s modulus *E_y_* [MPa]	732.060	740.260
Poisson’s ratio *ν*	0.164	−0.046

**Table 3:** Considered cases of inclusion patterns and phases combinations.

Case No.	Sample Inclusion Pattern	Inclusion Material	Matrix Material
1	3 vertical	Hex reentrant	Uniform honeycomb
2	3 vertical	Uniform honeycomb	Hex reentrant
3	3 vertical	Rotating rectangles unit	Orthogonal grid
4	3 vertical	Orthogonal grid	Rotating rectangles unit
5	3 horizontal	Hex reentrant	Uniform honeycomb
6	3 horizontal	Uniform honeycomb	Hex reentrant
7	3 horizontal	Rotating rectangles unit	Orthogonal grid
8	3 horizontal	Orthogonal grid	Rotating rectangles unit
9	9	Hex reentrant	Uniform honeycomb
10	9	Uniform honeycomb	Hex reentrant
11	9	Rotating rectangles unit	Orthogonal grid
12	9	Orthogonal grid	Rotating rectangles unit

**Table 4:** Geometrical parameters optimization range.

Sample Inclusion Pattern	Parameters Optimization Range [mm]					
	a	b	C	d	e	f
3 vertical rectangles	5–30	5–95	5–30	5–95	-	-
3 horizontal rectangles	5–95	5–30	5–95	5–30	-	-
9 rectangles	5–30	5–30	5–30	5–30	5–30	5–30

**Table 5:** Effective properties of obtained multiphase materials; 3 vertical inclusion pattern.

Case No.	Inclusion Regions Dimensions [mm]				Effective Young’s Modulus [MPa]	Effective Poisson’s Ratio
	a	b	c	d		
1	29.162	64.18	29.645	37.041	26.985	0.016
2	29.964	48.329	29.597	68.434	26.543	−0.072
3	29.009	86.731	29.723	89.297	740.130	−4.467 × 10^−5^
4	28.216	28.698	27.652	24.452	740.760	−4.164 × 10^−5^

**Table 6:** Effective properties of obtained multiphase materials; 3 horizontal inclusion pattern.

Case No.	Inclusion Regions Dimensions [mm]				Effective Young’s Modulus [MPa]	Effective Poisson’s Ratio
	a	b	c	d		
5	85.628	17.277	87.063	23.125	56.365	0.132
6	92.424	14.814	93.807	16.082	48.529	0.099
7	94.918	28.556	93.883	27.193	742.090	9.713 × 10^−6^
8	62.218	11.381	88.793	8.3644	742.960	1.928 × 10^−4^

**Table 7:** Effective properties of obtained multiphase materials; 9 rectangles inclusion pattern.

Case No.	Inclusion Regions Dimensions [mm]						Effective Young’s Modulus [MPa]	Effective Poisson’s Ratio
	a	b	c	d	e	f		
9	22.402	26.615	28.944	20.317	29.019	17.655	45.820	0.055
10	27.573	12.933	28.541	18.167	28.999	19.296	37.200	0.092438
11	29.435	27.364	29.95	28.943	29.812	29.676	741.080	–1.577 × 10^−5^
12	28.203	7.207	29.218	10.054	29.233	6.909	742.630	4.310 × 10^−5^

## Data Availability

The original contributions presented in this study are included in the article material. Further inquiries can be directed to the corresponding author.

## References

[1] Evans K.E. (1991). Auxetic polymers: A new range of materials. Endeavour.

[2] Fortes M.A., Teresa Nogueira M. (1989). The poison effect in cork. Mater. Sci. Eng. A.

[3] Love A.E. (1927). A Treatise on the Mathematical Theory of Elasticity.

[4] Voigt W. (1928). Lehrbuch der Kristallphysik.

[5] Bhullar S.K. (2015). Three decades of auxetic polymers: A review. e-Polymers.

[6] Lim T.C. (2015). Auxetic Materials and Structures.

[7] Evans K.E., Alderson A. (2000). Auxetic materials: Functional materials and structures from lateral thinking!. Adv. Mater..

[8] Elipe J.C.A., Lantada A.D. (2012). Comparative study of auxetic geometries by means of computer-aided design and engineering. Smart Mater. Struct..

[9] García-Aznar J.M., Nasello G., Hervas-Raluy S., Pérez M.A., Gómez-Benito M.J. (2021). Multiscale modeling of bone tissue mechanobiology. Bone.

[10] Horstemeyer M.F., Leszczynski J., Shukla M. (2009). Multiscale modeling: A review. Practical Aspects of Computational Chemistry.

[11] Meena K., Singamneni S. (2019). A new auxetic structure with significantly reduced stress concentration effects. Mater. Des..

[12] Cho H., Seo D., Kim D.N., Schmauder S., Chen C.S., Chawla K., Chawla N., Chen W., Kagawa Y. (2019). Mechanics of auxetic materials. Handbook of Mechanics of Materials.

[13] Wang T., Li Z., Wang L., Hulbert G.H. (2020). Crashworthiness analysis and collaborative optimization design for a novel crash-box with re-entrant auxetic core. Struct. Multidiscip. Optim..

[14] Ren X., Shen J., Tran P., Ngo T.D., Xie Y.M. (2018). Auxetic nail: Design and experimental study. Compos. Struct..

[15] Momoh E.O., Jayasinghe A., Hajsadeghi M., Vinai R., Evans K.E., Kripakaran P., Orr J. (2024). A state-of-the-art review on the application of auxetic materials in cementitious composites. Thin-Walled Struct..

[16] Gao Q., Lu Y., Shi Y., Liao W.-H., Yin G., Li J., Xiao F., Qiu R. (2023). Enhancing the output performance of energy harvesters using hierarchical auxetic structure and optimization techniques. IEEE Trans. Ind. Electron..

[17] Gohar S., Hussain G., Ilyas M., Ali A. (2021). Performance of 3D printed topologically optimized novel auxetic structures under compressive loading: Experimental and FE analyses. J. Mark. Res..

[18] Behinfar P., Nourani A. (2024). Analytical and numerical solution and multi-objective optimization of tetra-star-chiral auxetic stents. Discov. Appl. Sci..

[19] Bruggi M., Zega V., Corigliano A. Optimization of auxetic structures for MEMS applications. Proceedings of the 17th International Conference on Thermal, Mechanical and Multi-Physics Simulation and Experiments in Microelectronics and Microsystems (EuroSimE).

[20] Wang Z.P., Poh L.H., Zhu Y., Dirrenberger J., Forest S. (2019). Systematic design of tetra-petals auxetic structures with stiffness constraint. Mater. Des..

[21] Wang M., Sun S., Zhang T.Y. (2023). Machine learning accelerated design of auxetic structures. Mater. Des..

[22] Tan H., He Z., Li E., Cheng A., Chen T., Tan X., Li Q., Xu B. (2021). Crashworthiness design and multi-objective optimization of a novel auxetic hierarchical honeycomb crash box. Struct. Multidiscip. Optim..

[23] Novak N., Nowak M., Vesenjak M., Ren Z. (2022). Structural optimization of the novel 3D graded axisymmetric chiral auxetic structure. Phys. Status Solidi B.

[24] Meier T., Li R., Mavrikos S., Blankenship B., Vangelatos Z., Yildizdag M.E., Grigoropoulos C.P. (2024). Obtaining auxetic and isotropic metamaterials in counterintuitive design spaces: An automated optimization approach and experimental characterization. Comput. Mater..

[25] Ashby M.F., Bréchet Y.J.M. (2003). Designing hybrid materials. Acta Mater..

[26] Kromm F.X., Quenisset J.M., Harry R., Lorriot T. (2002). An example of multimaterials design. Adv. Eng. Mater..

[27] Long K., Du X., Xu S., Xie Y.M. (2016). Maximizing the effective young’s modulus of a composite material by exploiting the Poisson effect. Compos. Struct..

[28] Zawistowski M., Poteralski A. (2024). Parametric optimization of selected auxetic structures. Multiscale Multidiscip. Model. Exp. Des..

[29] Zawistowski M., Poteralski A. (2024). Development of a hybrid material with auxetic phase. Multiscale Multidiscip. Model. Exp. Des..

[30] Clarke D.J., Carter F., Jowers I., Moat R.J. (2023). An isotropic zero Poisson’s ratio metamaterial based on the aperiodic ‘hat’ monotile. Appl. Mater. Today.

[31] Cimolai G., Qin Q., Mageira P., Dayyani I. (2025). Mechanical characterization of a 3D large strain zero Poisson’s ratio helical metamaterial. Commun. Mater..

[32] Del Broccolo S., Laurenzi S., Scarpa F. (2017). AUXHEX—A Kirigami inspired zero Poisson’s ratio cellular structure. Compos. Struct..

[33] Subramanian R. (2010). Strength of Materials.

[34] ANSYS Material Designer User’s Guide. https://ansyshelp.ansys.com/public/Views/Secured/corp/v251/en/pdf/Material_Designer_Users_Guide.pdf.

